# SNHG15 enhances cisplatin resistance in lung adenocarcinoma by affecting the DNA repair capacity of cancer cells

**DOI:** 10.1186/s13000-023-01291-2

**Published:** 2023-03-02

**Authors:** Yong Li, Hui-Qin Huang, Zheng-Hui Huang, Nan-Ding Yu, Xiang-Li Ye, Mei-Chen Jiang, Li-Min Chen

**Affiliations:** 1 Department of Respiration Medicine, Fujian Medical University Union Hospital, No.29 Xin Quan Road, Fuzhou, 350000 Fujian China; 2 Fujian Provincial Key Laboratory of Medical Testing, Fujian Academy of Medical Sciences, Fuzhou, 350000 Fujian China; 3 Department of Pathology, Fujian Medical University Union Hospital, Fuzhou, 350000 Fujian China

**Keywords:** SNHG15, E2F1, ECE2, Lung adenocarcinoma, Chemotherapy resistance

## Abstract

**Background:**

Lung adenocarcinoma (LUAD) is a prevalent malignancy. SNHG15 has been demonstrated to be oncogenic in many kinds of cancers, however the mechanism of SNHG15 in LUAD cisplatin (DDP) resistance remains unclear. In this study, we demonstrated the effect of SNHG15 on DDP resistance in LUAD and its related mechanism.

**Methods:**

Bioinformatics analysis was adopted to assess SNHG15 expression in LUAD tissues and predict the downstream genes of SNHG15. The binding relationship between SNHG15 and downstream regulatory genes was proved through RNA immunoprecipitation, chromatin immunoprecipitation and dual-luciferase reporter assays. Cell counting kit-8 assay was adopted to evaluate LUAD cell viability, and gene expression was determined by Western blot and quantitative real-time polymerase chain reaction. We then performed comet assay to assess DNA damage. Cell apoptosis was detected by Tunnel assay. Xenograft animal models were created to test the function of SNHG15 in vivo.

**Results:**

SNHG15 was up-regulated in LUAD cells. Moreover, SNHG15 was also highly expressed in drug-resistant LUAD cells. Down-regulated SNHG15 strengthened the sensitivity of LUAD cells to DDP and induced DNA damage. SNHG15 could elevate ECE2 expression through binding with E2F1, and it could induce DDP resistance by modulating the E2F1/ECE2 axis. In vivo experiments verified that the SNHG15 could enhance DDP resistance in LUAD tissue.

**Conclusion:**

The results suggested that SNHG15 could up-regulate ECE2 expression by recruiting E2F1, thereby enhancing the DDP resistance of LUAD.

**Supplementary Information:**

The online version contains supplementary material available at 10.1186/s13000-023-01291-2.

## Introduction

Lung cancer is one of the most prevalent cancers. Statistics in 2018 reported that lung cancer accounted for 11.6% of all the cancers worldwide [1]. The early symptoms of lung cancer are not obvious, and metastasis usually occurs before initial diagnosis [2]. Therefore, the mortality rate of lung cancer is extremely high, and its 5-year overall survival rate only reaches 16.6% [3]. Lung adenocarcinoma (LUAD) mainly occurs in the peripheral airways of the lung, which is the most common subtype of lung cancer [4]. Chemotherapy is a significant therapeutic strategy for lung cancer that is usually conducted with the combination of platinum and other chemotherapeutic agents. Cisplatin (DDP) is the most regular platinum drug [5]. Since DDP is the most frequently-used strategy in treating lung cancer, and for lung cancer patients, DDP resistance is also one of the leading causes for patients’ poor prognoses. DDP resistance will lead to cancer recurrence, metastasis or a delay in treatment, resulting in poor treatment efficacy [6]. Therefore, the focus about the future research of chemotherapy agents lies on investigating the drug resistance mechanism of LUAD and the interaction among the key regulatory targets, and then developing effective treatment regimen, thereby improving the treatment outcome of patients.

LncRNAs, exerting regulatory effect on gene expression, participate in multiple pathological and physiological processes, such as tumor genesis and progression [7]. Altered expression of lncRNAs exerts great effect on modulating the progression of cancers, including LUAD. LncRNA SNHG15 is located on chromosome 7p13 [8], which has been demonstrated to be up-regulated in hepatocellular carcinoma [9], LUAD [10] and breast cancer [8]. Its overexpression can promote cancer cell proliferation, migration and invasion. Moreover, studies demonstrated that SNHG15 exerted a crucial effect on chemotherapeutic resistance. For example, SNHG15 could induce DDP resistance in breast cancer by activating miR-381 [8]. SNHG15 enhanced doxorubicin resistance through miR-381-3p/GFRA1 axis in osteosarcoma [11]. Qu et al. [12] found a higher expression of SNHG15 in DDP-resistant epithelial ovarian cancer cells compared to the control group, suggesting that up-regulating SNHG15 enhanced the drug resistance of epithelial ovarian cancer cells. Therefore, we assumed that SNHG15 was related to DDP resistance in LUAD cells, and we intended to clarify the molecular mechanism by which SNHG15 enhances DDP resistance in LUAD cells.

E2F is a group of transcription factors (TF) that exerts important effect on cell proliferation, differentiation and apoptosis [13]. E2F1 is the first identified member in E2F family. It is well-recognized that TF E2F1 can modulate cell cycle, and work as a mediator for DNA damage-induced apoptosis and checkpoint responses [14, 15]. Plenty of studies have reported the tumor-promoting effect of E2F1. For example, overexpression of E2F1 enhanced the resistance of paclitaxel combined with DDP and accelerated gastric cancer cell proliferation, while silenced E2F1 promoted the treatment efficacy of paclitaxel combined with DDP [16]. Guo et al. [17] demonstrated that the lncRNA HAGLR suppressed the development of LUAD through recruiting DNMT1 and suppressing E2F1 expression. Through bioinformatics analysis, we found that E2F1 might be a downstream regulator of SNHG15, and we intended to determine the exact regulatory relationship between them.

Endothelin Converting Enzyme 2 (ECE2), an enzyme involved in neuropeptide production [18], is mainly located on the cytoplasm of the membrane [19], which is also considered to be a type of antigen for autoimmune polyendocrine syndrome type 1 [20]. It has been reported that ECE2 is involved in the processing of various neuroendocrine peptides, such as angiotensin I, P, and preenkephalin-derived peptides, and it may also play a role in the processing of amyloid β. However, its expression in LUAD and its effect on LUAD cells remains unclear.

This study revealed the expression pattern, function and molecular mechanism of SNHG15 in LUAD DDP resistance. We performed bioinformatics analysis and found the up-regulation of SNHG15 in LUAD tissues and cells, particularly in the resistant cells. Afterwards, a series of cell and animal experiments were conducted, the results of which suggested that SNHG15 enhanced DDP resistance in LUAD through the E2F1/ECE2 axis. Our study indicated that the SNHG15/E2F1/ECE2 axis could affect the DDP resistance in LUAD cells, which offered a new insight for the improvement of LUAD therapy and the development of related drugs.

## Materials and methods

### Bioinformatics analysis

Based on TCGA-LUAD database (https://portal.gdc.cancer.gov/), data of LUAD gene expression (535 cancer samples and 59 normal samples) and related clinical data were obtained. SNHG1, highly expressed in LUAD, was selected for further study. The downstream TFs of SNHG15 were predicted by lncMAP database (http://bio-bigdata.hrbmu.edu.cn/LncMAP/survival.jsp). According to the strong correlation, E2F1/ECE2 was chosen for our study. Meanwhile, RNA–Protein Interaction Prediction (RPISeq) (http://pridb.gdcb.iastate.edu/RPISeq/index.html) was used to evaluate the possibility of interaction between lncRNA and TF. According to the median expression of ECE2, the target gene of E2F1, we divided the cancer samples into two groups, namely, high and low expression group. Then, survival analysis was conducted by using the survival package. Next, we used JASPAR database (http://jaspar.genereg.net/) for predicting the binding sequence of the TF and the target gene promoter region (2000 bp upstream of the target gene transcription start site was selected as the promoter region).

### Cell culture

LUAD cell lines NCI-H1299 (CRL-5803™), NCI-H1975 (CRL-5908™), A549 (CRM-CCL-185™) and human normal lung epithelial cell line BEAS-2B (CRL-9609™) were obtained from American Type Culture Collection (USA). A549-WT was DDP-sensitive and written as A549/CS. H1975-WT was DDP-sensitive and written as H1975/CS. H1299-WT was DDP-sensitive and written as H1299/CS. The above cells were cultured in Dulbecco’s modified Eagle medium (DMEM) medium with 10% fetal bovine serum (FBS), and then we incubated the medium in an incubator at 37 ℃ with 5% CO_2_.

DDP-resistant A549 cells (A549/CR, BNCC341254, BeNa Culture Collection, China), DDP-resistant H1975 cells (H1975/CR) and DDP-resistant H1299 cells (H1299/CR) were cultured. The three DDP-resistant cell lines were cultured in DMEM containing 10% FBS and placed in an incubator with 5% CO_2_ at 37 ℃.

### Cell counting kit-8 (CCK-8) assay

We conducted the CCK-8 assay for determining the sensitivity of A549/CR, A549/CS, H1975/CR, H1975/CS, H1299/CR and H1299/CS cells to DDP. We seeded the cells and cultured them in 96-well plates (5 × 10^3^ cells/well) for 24 h, and then we treated the cells with DDP of different concentrations (0, 0.5, 1, 2, 4, 6, 8, 10, 12 μg/mL) [21]. After 24 h, we added 10 μL of CCK-8 (Dojindo Laboratories, Japan) to each well and cells were incubated for 2 h at 37 ℃. The OD value at 450 nm was detected, and the median inhibitory concentration (IC_50_) was calculated.

### Quantitative real-time polymerase chain reaction (qRT-PCR)

Referring to the manufacturer's protocol, we extracted total RNA from cells using TRIzol® reagent (Thermo Fisher Scientific, USA). We then reversely transcribed the RNA into cDNA by PrimeScript™ kit (Takara Biotechnology, Japan). SYBR® Green PCR Master Mix (Applied Biosystems, USA) was applied in PCR amplification of cDNA products. Glyceraldehyde 3-phosphate dehydrogenase was taken as the internal reference, the gene expression levels were determined by the 2^−ΔΔCt^ method. We presented the primer sequences in Table 1.

**Table 1:** Primer sequences for qRT-PCR

Gene	Primer sequence (5’ → 3’)
SNHG15	F: GCTGAGGTGACGGTCTCAAA
	R: GCCTCCCAGTTTCATGGACA
ECE2	F: AATGAAATCGTCTTCC
	R: GTCAGTGACTCATTCT
GAPDH	F: GGTCTCCTCTGACTTCAACA
	R: AGCCAAATTCGTTGTCATAC

### Comet assay

We pre-coated the slides by using 1% normal melting agarose. Approximately 3 × 10^4^ cells (A549/CR, H1975/CR and H1299/CR) treated with DDP (5 μg/mL) and 70 μL 1% low melting point agarose were mixed in phosphate buffered saline (PBS) and rapidly spread onto pre-coated slides. We then promptly transferred the slides into cold lysis buffer consisting of 100 mM ethylenediaminetetraacetic acid, 1% Triton X-100, 2.5 M NaCl, 10 mM Tris (pH 7.5), 1% hydrogen chloride, and 10% dimethyl sulfoxide at 4 ℃ for 1 to 3 h. The slides were then put into electrophoresis solution for 20 min to promote DNA unwinding, followed with 20 min of electrophoresis at 25 V and 300 mA. Afterwards, we cleared the slides with PBS and then stained them by propidium iodide. Individual cell was observed through an Olympus BX51 UV fluorescence microscope (Olympus, Japan) [22].

### Terminal deoxynucleotidyl transferase-mediated dUTP-biotin nick end labeling (TUNEL) assay

The TUNEL fluorescence FITC kit (Roche, Switzerland) was applied to evaluate apoptosis. We referred to the manufacturer’s instructions to carry out this assay, and the cells were assessed by using a laser confocal microscope. We presented the amount of apoptosis as its proportion of total cells [23].

### Immunofluorescence analysis

Coverslips of four-well slides were used for cell culture. Then 95% ethanol was applied for fixation of the coverslips and 0.1% Triton X-100 was adopted for permeabilization. Then goat serum (ZSGB-Bio, China) was applied to block the cells which were incubated with anti-mouse γH2AX (CST, USA) antibody. We then incubated the cells with the secondary antibody, and 1 h later, we used DAPI to counterstain them. A confocal microscope was applied for imaging (Leica, Germany) [24].

### Western blot

Lysate was added to the cells to extract the total proteins which were then isolated through sodium dodecyl sulfate polyacrylamide gel electrophoresis. Afterwards, we transferred the protein onto polyvinylidene fluoride membranes, followed by 2-h blockage with 5% skimmed milk. We incubated the membranes at 4 ℃ overnight with primary antibodies anti-rabbit ECE2 (Thermo Fisher Scientific, USA) and anti-rabbit GADPH (Abcam, UK). Next, we incubated them for 2 h with goat anti-rabbit secondary antibody (Abcam, UK). Gel imaging system and enhanced chemiluminescence substrate kit (Thermo Fisher Scientific, USA) were used to assess the protein expression.

### Fluorescence in situ hybridization (FISH) assay

We conducted the RNA in situ hybridization through using lncRNA FISH Probe Mix Kit (RiboBio, China). We then seeded the cells in laser-confocal dishes, followed by 10 min fixation in 4% paraformaldehyde, and 5 min permeabilization with 0.5% Triton X-100, next we used PBS to wash them 3 times at room temperature. Cells were then soaked in prehybridization buffer at 37 ℃ for 0.5 h and incubated overnight at 37 ℃ with target probe hybridization buffer. We washed the cells with sodium citrate buffer and counterstained them with DAPI. Images were ultimately evaluated through confocal microscopy [25].

#### Dual-luciferase assay

We developed luciferase reporter vectors of mutant and wild type ECE2. Then we co-transfected them with overexpressed E2F1 and its negative control plasmids into A549/CR cells. The luciferase activity was determined by dual-luciferase reporter assay system (Promega, USA).

#### RNA Immunoprecipitation (RIP) assay

We conducted RIP assay through using E2F1 antibody and normal mouse IgG antibody for negative control. The exact procedures referred to the manufacturer’s instruction for Magna RIP kit (Millipore, USA). We incubated the cell lysates with magnetic beads bound with antibodies in RIP buffer and the final precipitated RNA was isolated for the use of qRT-PCR analysis.

#### Chromatin immunoprecipitation (ChIP) assay

We obtained chromatin fragments in 200–500 bp through sonicating the cells in formaldehyde. We then performed immunoprecipitation separately with anti-E2F1 and anti-IgG antibodies, and chromatin DNA was extracted and assessed through qRT-PCR. We listed the applied primer sequences in Table 2.

**Table 2:** Primer sequences for ChIP-qPCR

Primer Sets	Primer sequence (5’ → 3’)
Primer pair	F: TACATTCTGTGGGCATCTCGT
	R: GCAGTTCACTCACATTCAGGG

#### Construction of xenograft tumor

12 BALB/c mice (8–10 g, 4 weeks) were housed in specific pathogen Free environment. The subcutaneous inoculation was conducted on the mice with A549/CR cells (1 × 10^6^, 200 μL) which were stably transfected by sh-SNHG15 or sh-NC. Tumor volume was evaluated by caliper every 4 days when tumors grew to visible size and statistics were calculated as (length × width^2^)/2. After 28 days we sacrificed the mice, from which the tumors were obtained and weighed. We stored a part of tumor tissues at -80 ℃ for qRT-PCR and the rest of them were placed in formalin for Immunohistochemistry (IHC). All animal experiments were approved by the Animal Experimentation Ethics Committee of Fujian Medical University Union Hospital.

#### IHC assay

We fixed tumor tissues with 10% paraformaldehyde and embedded them in paraffin. We performed serial section on tumors (4 μm thickness) and then immunohistochemically stained them. After antigen retrieval, sections were incubated overnight with ECE2, γH2AX antibodies (Thermo Fisher Scientific, USA) and then incubated with secondary antibodies for 1 h. Afterwards, we stained the specimens by using DAB and hematoxylin. After that, the specimens were dehydrated, cleaned, mounted, and observed and photographed with a microscope [26].

#### Data analysis

We presented the data as mean ± standard deviation, and the assays were repeated 3 times. We chose GraphPad Prism 6.0 for the analysis of all the results. Next, we separately applied one-way analysis of variance and t-test to test differences among multiple groups and between two groups. *P* < 0.05 was considered statistically significant.

## Results

### SNHG15 expression is up-regulated in LUAD

**Fig. 1:**
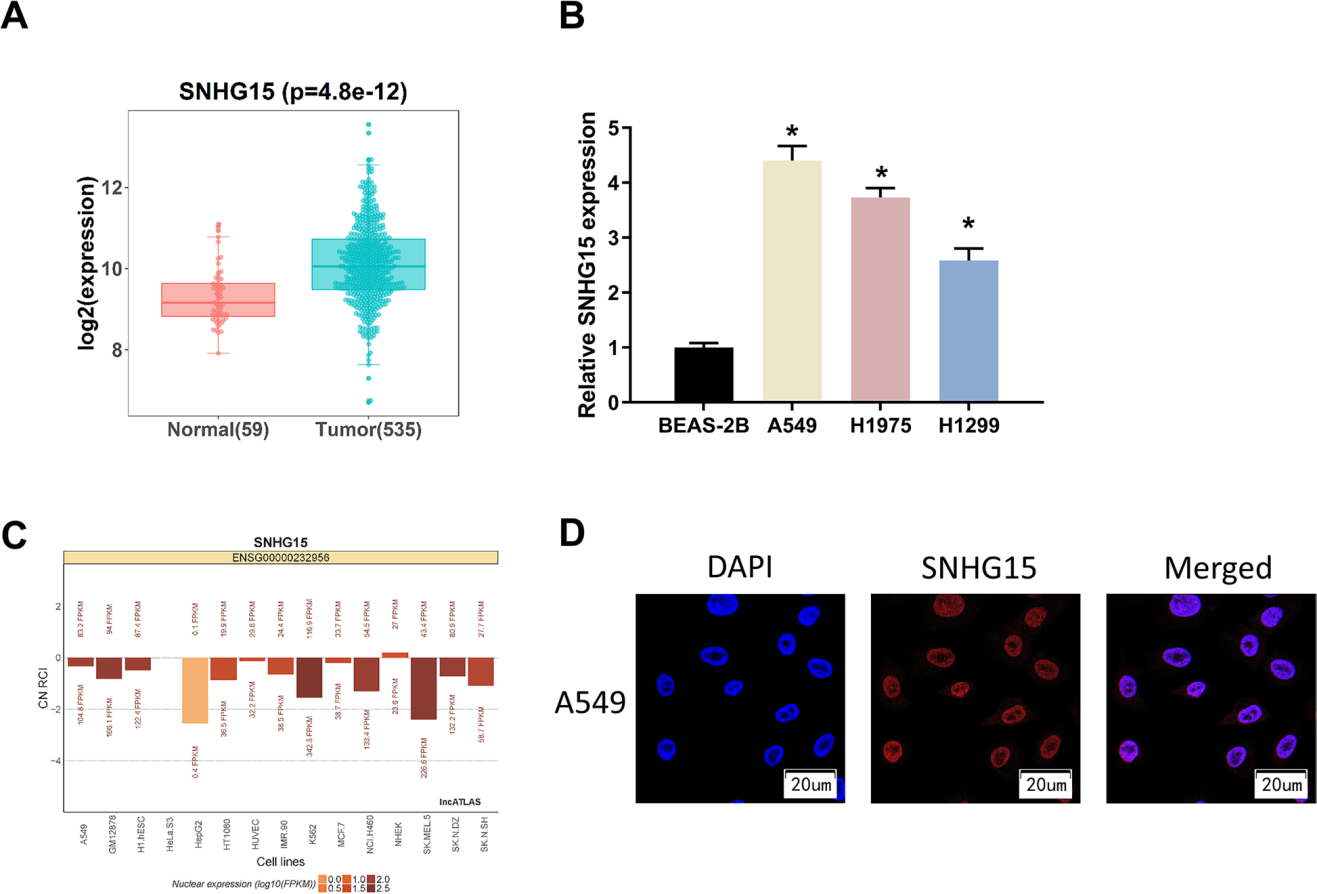
SNHG15 expression is up-regulated in LUAD cells. **A** Box plots of SNHG15 expression analyzed based on TCGA-LUAD database, with red indicating normal tissues and green indicating tumor tissues. **B** The expression levels of SNHG15 in human normal lung epithelial cells BEAS-2B and LUAD cells A549, H1975 and H1299 assessed by qRT-PCR. **C** Localization map of SNHG15 subcellular predicted by bioinformatics analysis. **D** FISH assay verified SNHG15 subcellular localization. (* indicates *P* < 0.05)

It has been documented that SNHG15 works as an oncogene to accelerate the progression of many cancers including colorectal cancer and hepatocellular carcinoma [9, 27], but it has rarely been reported in LUAD. We screened SNHG15 as the target of our study and investigated its regulatory mechanism in LUAD. According to the results of TCGA-LUAD database analysis, we demonstrated a markedly high expression of SNHG15 in LUAD tissues (Fig. 1A), and qRT-PCR analysis of SNHG15 expression in LUAD cells yielded a consistent result (Fig. 1B). We then predicted the subcellular localization of SNHG15 through bioinformatics analysis, which indicated that SNHG15 was expressed and functioned as a regulatory factor in the nucleus (Fig. 1C). FISH assay also verified the above result (Fig. 1D). As the results presented, we demonstrated the high expression of SNHG15 in LUAD, and it was localized in the nucleus of LUAD cells.

### SNHG15 enhances DDP resistance in LUAD cells

**Fig. 2:**
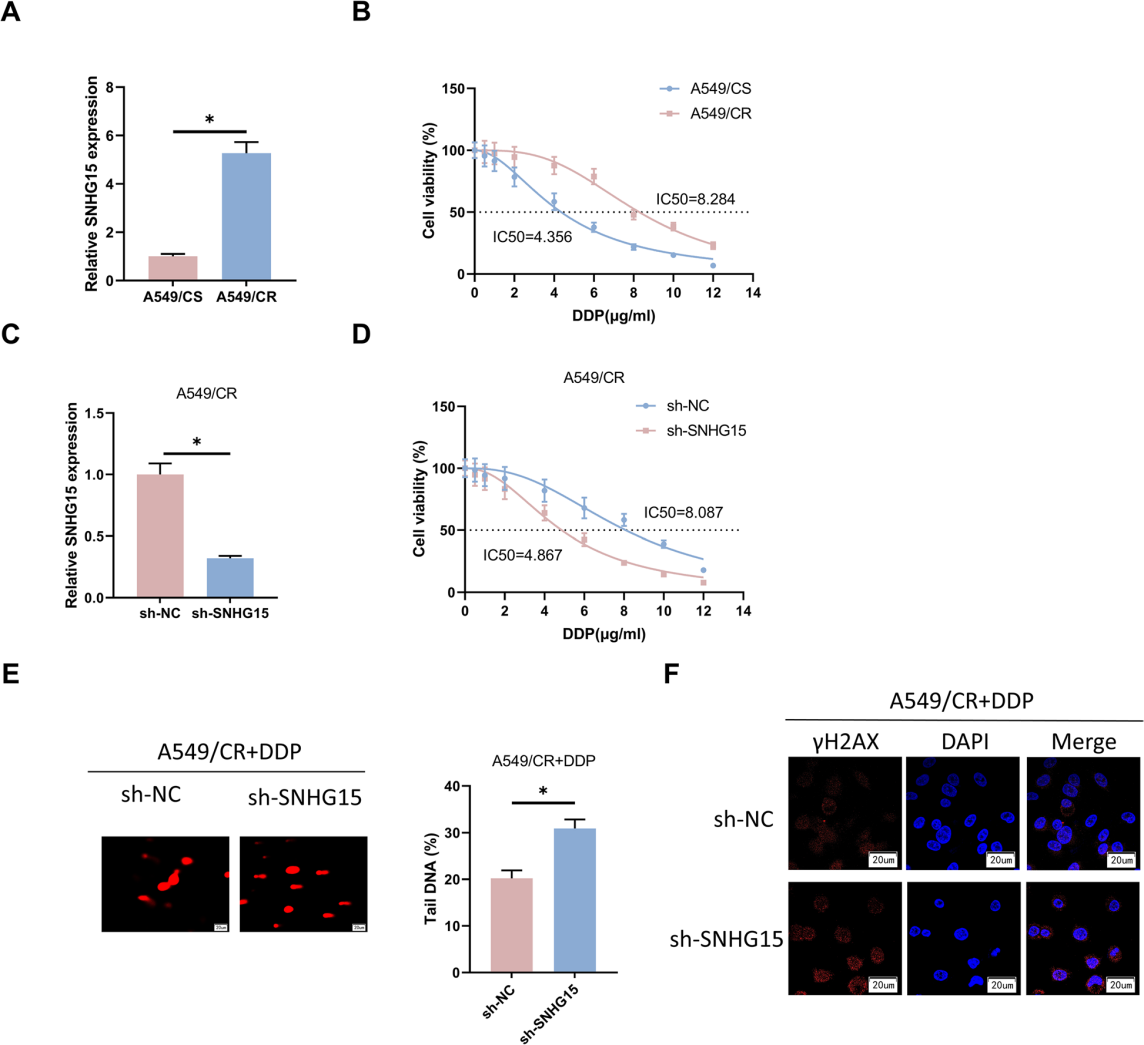
SNHG15 enhances DDP resistance in A549/CR cells. **A** SNHG15 expression in A549/CS and A549/CR cells assessed by qRT-PCR. **B** IC_50_ values of DDP in A549/CS and A549/CR cells evaluated through CCK-8 assay. **C** SNHG15 expression in A549/CR cells transfected with sh-NC and sh-SNHG15 determined by qRT-PCR. **D** IC_50_ values of DDP in A549/CR cells transfected with sh-NC and sh-SNHG15 evaluated through CCK-8 assay. **E** DNA damage in DDP-treated (3 µg/mL) A549/CR cells transfected with sh-NC and sh-SNHG15 observed by comet assay. (Scale bar: 20 µm) (**F**) Immunofluorescence assay was performed to assess γH2AX production in DDP-treated (3 µg/mL) A549/CR cells transfected with sh-NC and sh-SNHG15, and photographs were taken by using confocal microscopy. (Scale bar: 20 µm) (* indicates *P* < 0.05)

To reveal the mechanism of SNHG15 resistance in LUAD, we selected DDP-resistant cells A549/CR, DDP-sensitive cells A549/CS, DDP-resistant cells H1975/CR, DDP-sensitive cells H1975/CS, DDP-resistant cells H1299/CR and DDP-sensitive cells H1299/CS. Afterwards, we examined SNHG15 expression in the LUAD cell lines through qRT-PCR and the results showed a high expression of SNHG15 in DDP-resistant cells (Fig. 2A, Supplementary Fig. 1A, 2A). The results indicated that the drug-resistant cell lines were constructed and could be used for subsequent experiments. CCK-8 assay demonstrated a higher IC_50_ value of DDP in DDP-resistant cells than in DDP-sensitivity cells (Fig. 2B, Supplementary Fig. 1B, 2B). Subsequently, we transfected sh-NC and sh-SNHG15 respectively into A549/CR cells, so as to investigate the role of altered SNHG15 expression in DDP resistance of LUAD cells. The transfection efficiency was verified by qRT-PCR, which presented that SNHG15 was notably lowly expressed in sh-SNHG15 group (Fig. 2C). The IC_50_ of DDP after SNHG15 knockdown was markedly lower compared to the control group (Fig. 2D), which indicated that knockdown of SNHG15 could suppress DDP resistance in A549/CR cells. In summary, SNHG15 exerted a crucial effect on DDP resistance in LUAD cells.

Since DDP is a chemotherapeutic agent that exerts killing effect through DNA damage, and the DNA damage repair ability of tumor cells is a key factor in inducing DDP resistance. To reveal the mechanism of SNHG15 accelerating drug resistance in A549/CR cells, we performed comet assay to determine the role of SNHG15 in the DNA damage repair ability of A549/CR cells. The results indicated that down-regulating SNHG15 could dramatically enhance DDP-induced DNA damage (Fig. 2E). Next, we evaluated the DNA damage in accordance with the expression of γH2AX. Immunofluorescence analysis revealed an increase in the amount of γH2AX in DDP-treated A549/CR cells transfected with sh-SNHG15 by comparing to the control group (Fig. 2F), indicating that knockdown of SNHG15 accelerated DNA damage. In conclusion, SNHG15 could suppress the DDP sensitivity of LUAD cells through attenuating DNA damage.

### SNHG15 up-regulates ECE2 expression by recruiting E2F1

**Fig. 3:**
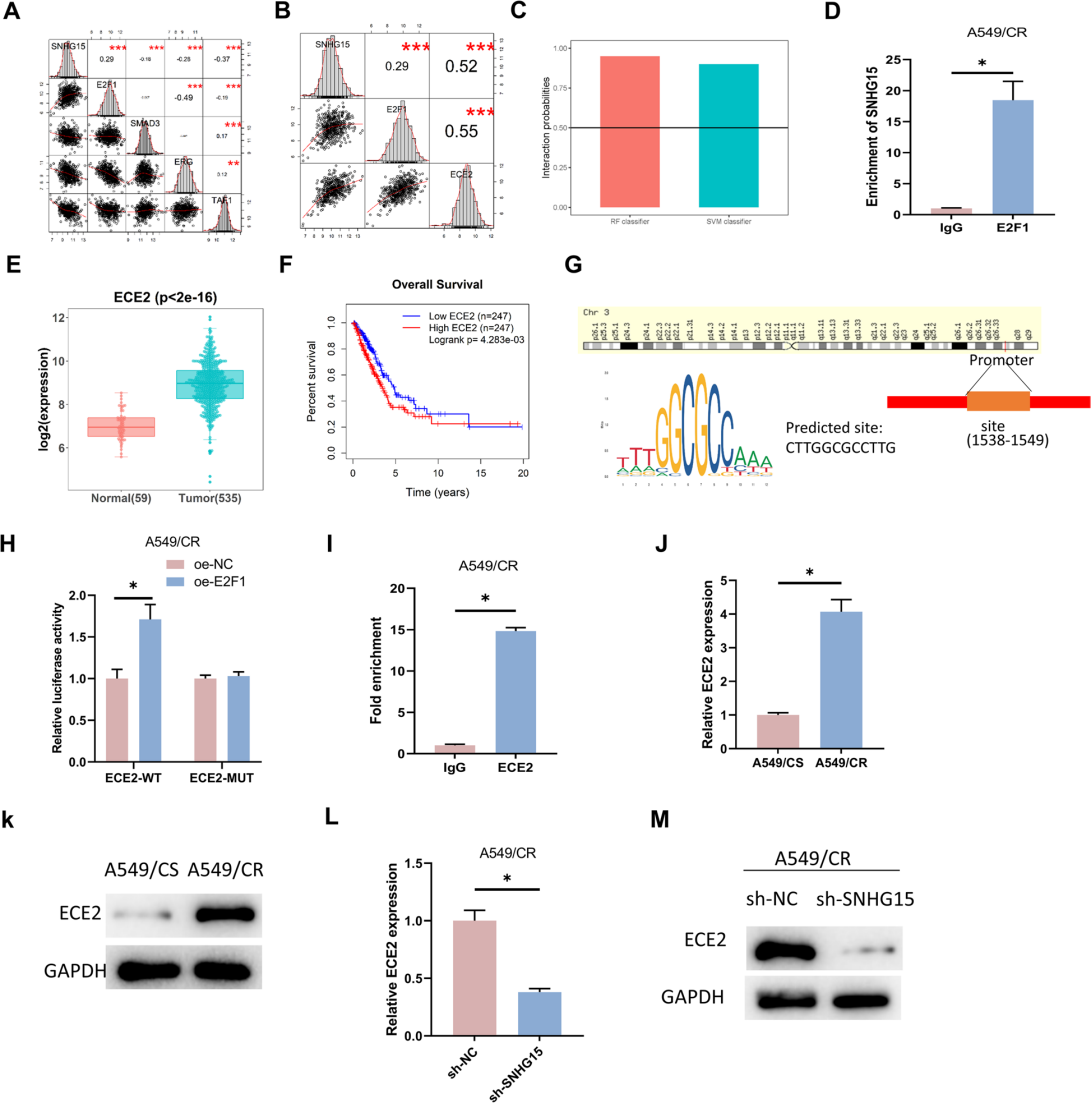
SNHG15 up-regulated ECE2 expression through recruiting E2F1. **A** Correlation analysis between SNHG15 and downstream transcription factors. **B** Correlation analysis of SNHG15 with E2F1 and ECE2. **C** Protein interaction score of SNHG15 and E2F1 predicted through RPISeq database. **D** The binding ability of SNHG15 and TF E2F1 verified through RIP assay. **E** Box plot of ECE2 expression in LUAD, red indicated normal tissues and green indicated tumor tissues. **F** Survival analysis of LUAD patients with high and low ECE2 expression. **G** JASPAR database was applied for predicting the binding sequences of ECE2 promoter region and E2F1. **H**, **I** Dual-luciferase and ChIP assays was performed to determine the binding of E2F1 and ECE2 promoter region. **J**, **K** ECE2 expression in A549/CS and A549/CR cell lines assessed through qRT-PCR and Western blot. **L**, **M** ECE2 expression in A549/CR cells transfected with sh-NC and sh-SNHG15 evaluated through qRT-PCR and Western blot. (* indicates *P* < 0.05)

LncMAP database was applied to predict SNHG15 triplet status in LUAD. We conducted correlation analysis on the predicted TFs and the target mRNA with SNHG15. The results showed that E2F1 was positively correlated with SNHG15 with the highest correlation, while TAF1 was negatively correlated with SNHG15 with the highest correlation (Fig. 3A). The triplet data analysis showed that ECE2 was the only candidate target gene for E2F1, and the expression of ECE2 was significantly positively correlated with that of E2F1(Fig. 3B). Therefore, E2F1 and ECE2 were selected as the downstream TF. Previous studies have reported that E2F1 can work as a transcriptional activator to accelerate cell proliferation and migration [28–30]. Meanwhile, scores from RPISeq database indicated that the valuations of SVM classifier and RF classifier were greater than 0.5 (Fig. 3C) [31, 32], which suggested high credibility for the interaction between SNHG15 interacted and E2F1. The RIP results showed a dramatically enhanced SNHG15 enrichment with the presence of E2F1 compared to IgG (Fig. 3D). The above assays demonstrated that SNHG15 could exert a downstream regulatory effect through recruiting TF E2F1.

Analysis of the triplet downstream data revealed that ECE2 was markedly highly expressed in cancer tissues (Fig. 3E). High ECE2 expression led to a shorter survival time for LUAD patients (Fig. 3F). The binding score of ECE2 promoter region and E2F1 was predicted to be > 10 in accordance with the JASPAR database, and the binding sequences were listed in Fig. 3G. To verify the binding relationship between them, we constructed wild- and mutant-type ECE2 sequences for dual-luciferase assay. We found the luciferase activity of the wild-type ECE2 promoter region enhanced notably after E2F1 overexpression, while there was no striking change in the mutant-type group (Fig. 3H). It was demonstrated in ChIP assay that the enrichment of ECE2 was markedly stimulated with the presence of E2F1 compared to IgG (Fig. 3I). The above assays determined the binding relationship between E2F1 and ECE2.

We then examined the relationship between ECE2 and DDP resistance in LUAD cells. We performed qRT-PCR and Western blot for determining the ECE2 expression in A549/CS and A549/CR cell lines. We found that ECE2 was dramatically up-regulated in A549/CR cells (Fig. 3J-K). Finally, we assessed the role of SNHG15 in ECE2 expression and demonstrated that knockdown of SNHG15 markedly attenuated ECE2 expression by comparing with the control group (Fig. 3L-M). It was suggested that SNHG15 up-regulated ECE2 expression through recruiting E2F1.

### SNHG15/ E2F1/ECE2 axis enhances DDP resistance in LUAD cells

**Fig. 4:**
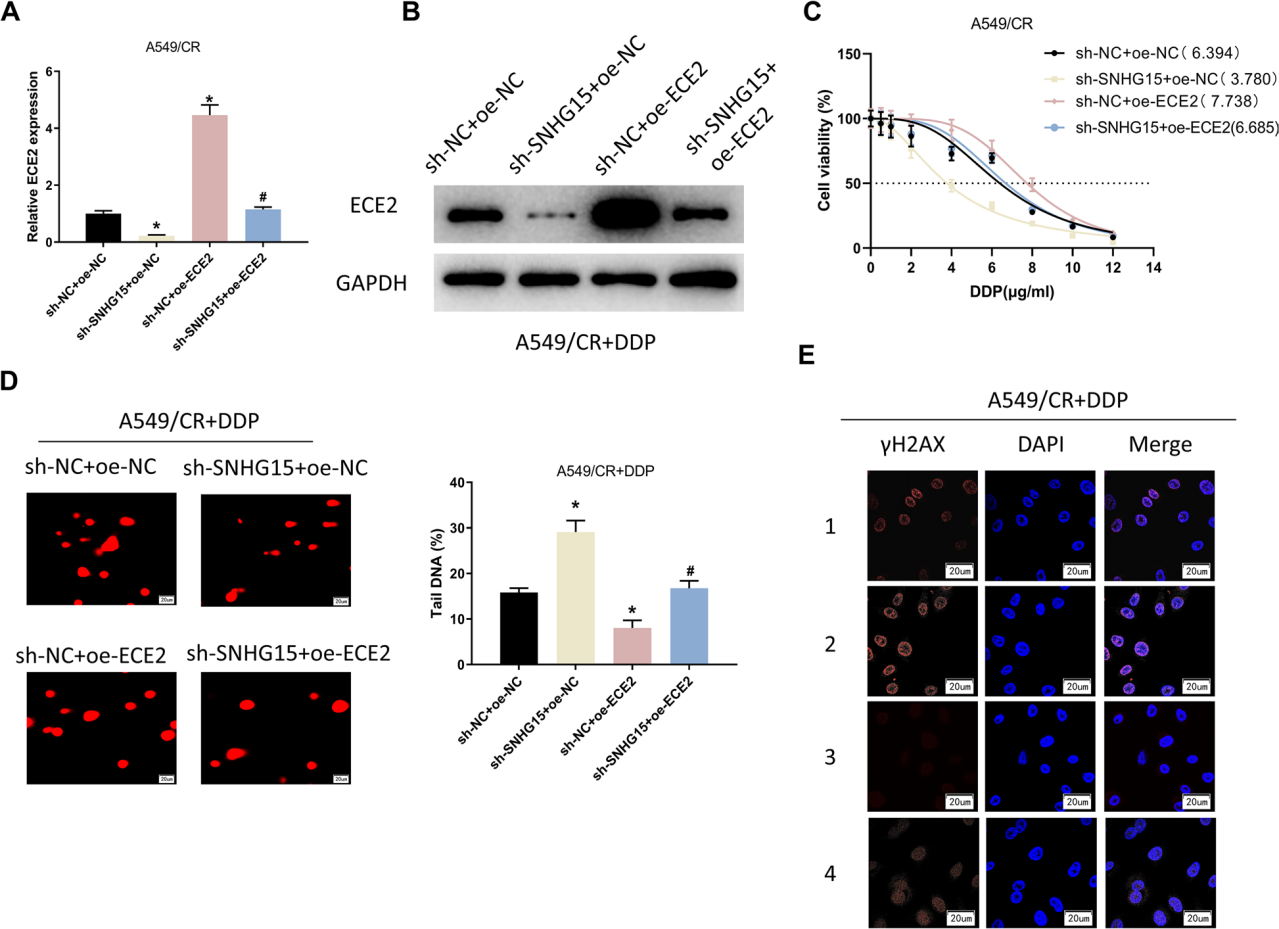
SNHG15/ E2F1/ECE2 axis enhances DDP resistance in LUAD cells. **A**, **B** ECE2 expression in different treatment A549/CR cells assessed by qRT-PCR and Western blot. (**C**) Cell viability and IC_50_ values of different treatment A549/CR cells determined through CCK-8. **D** DNA damage in different treatment A549/CR cells observed through comet assay (scale bar: 20 µm). **E** Immunofluorescence assay was applied to evaluate the production of γH2AX in different treatment DDP-treated (3 µg/mL) A549/CR cells transfected with sh-NC and sh-SNHG15. Photographs were taken through using confocal microscopy. 1: sh-NC + oe-NC; 2: sh-SNHG15 + oe-NC; 3: sh-NC + oe-ECE2; 4: sh-SNHG15 + oe-ECE2 (scale bars: 20 µm). (*/# indicates *P* < 0.05)

To reveal the resistance mechanism of SNHG15/ E2F1/ECE2 axis in LUAD, we constructed four cell lines based on A549/CR cells, H1975/CR cells and H1299/CR cells including sh-SNHG15 + oe-ECE2, sh-NC + oe-ECE2, sh-SNHG15 + oe-NC and sh-NC + oe-NC. The transfection efficiency was subsequently assessed by qRT-PCR and Western blot. We found that ECE2 expression was dramatically attenuated in sh-SNHG15 + oe-NC group and enhanced in sh-NC + oe-ECE2 group by comparing to the control group. The ECE2 expression was reverted in sh-SNHG15 + oe-ECE2 group (Fig. 4A-B, Supplementary Fig. 1C-D, 2C-D). CCK-8 assay demonstrated that knockdown of SNHG15 decreased IC_50_ values of DDP in A549/CR cells, H1975/CR cells, H1299/CR cells, and the IC_50_ values were upregulated after ECE2 overexpression. ECE2 overexpression reversed the effect of SNHG15 knockdown on IC_50_ values in the cells (Fig. 4C, Supplementary Fig. 1E, 2E). To verify the effect of SNHG15/ E2F1/ECE2 axis on DNA damage in A549/CR cells, H1975/CR cells and H1299/CR cells, we performed comet assay. The results showed that the DNA migration distance in DDP-treated A549/CR cells, H1975/CR cells and H1299/CR cells with knockdown of SNHG15 was markedly increased than that in the sh-NC + oe-NC group. In the meanwhile, the migration distance of DNA became shorter after overexpression of ECE2, which could reverse the effect of SNHG15 knockdown on DNA damage (Fig. 4D, Supplementary Fig. 1F, 2F). The results of γH2AX expression detected by immunofluorescence assay were consistent with those of comet assay (Fig. 4E, Supplementary Fig. 1G, 2G). In summary, SNHG15/E2F1/ECE2 axis enhances DDP resistance in LUAD cells.

### SNHG15 enhances DDP resistance in LUAD mouse model

**Fig. 5:**
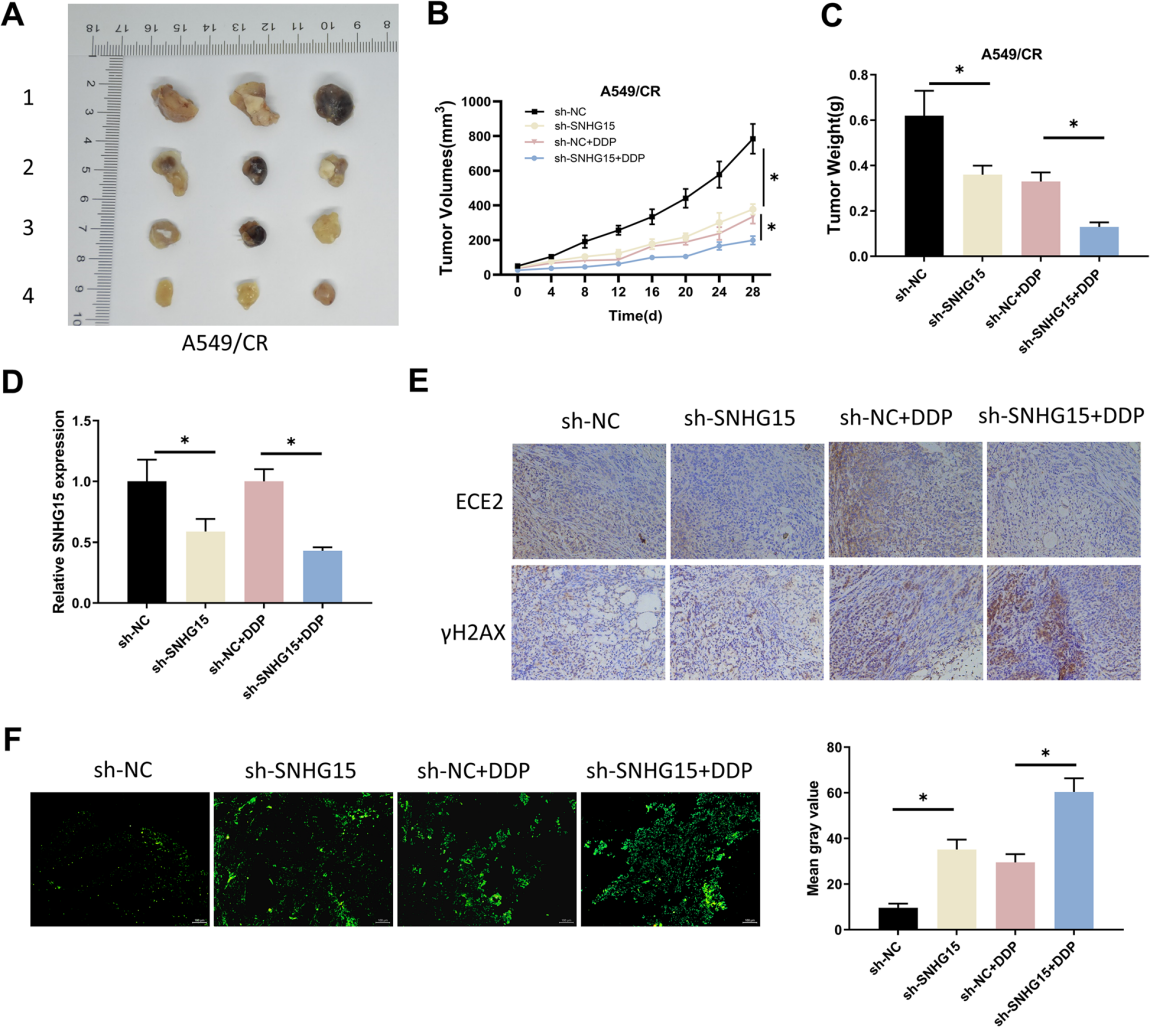
SNHG15 enhances DDP resistance in LUAD mouse model. **A** Size of tumors in BALB/C mice with different treatments. 1: sh-NC; 2: sh-SNHG15; 3: sh-NC + DDP; 4: sh-SNHG15 + DDP. **B** Tumor size measured every 4 days after different treatments; **C** Weight of mouse tumors after different treatments. **D** SNHG15 expression assessed through qRT-PCR with different treatments. **E** IHC assay was performed to determine the expression of ECE2 and γH2AX with different treatments. **F** Apoptosis in tumor sections measured through TUNEL assay (scale bar: 100 µm), and the mean gray value indicated the degree of apoptosis. (* indicates *P* < 0.05)

We constructed immunodeficient BALB/C mouse xenograft models through using cells stably transfected with sh-SNHG15 or sh-NC. We divided the mice into 4 groups: sh-SNHG15, sh-SNHG15 + DDP, sh-NC + DDP and sh-NC. Tumor size was subsequently measured periodically, and the results demonstrated that sh-SNHG15 treatment slowed tumor growth. In addition, tumor growth was suppressed with the treatment of DDP and sh-SNHG15 enhanced the efficacy of DDP (Fig. 5A-B). Tumor weight dropped with sh-SNHG15 treatment or DDP treatment, which was more pronounced with the combination of above treatments (Fig. 5C). These results indicated that knockdown of SNHG15 reduced the resistance of A549/CR cells to DDP. We performed qRT-PCR to assess the SNHG15 expression in tumor tissues, and found that sh-SNHG15 treatment down-regulated SNHG15 in mice without or with DDP treatment (Fig. 5D). We performed IHC assays to determine ECE2 and γH2AX expression. The results demonstrated that ECE2 was expressed in cytoplasm, while γH2AX was expressed in nucleus. After single sh-SNHG15 treatment, the expression level of ECE2 was decreased, while γH2AX expression was increased. After single DDP treatment, ECE2 expression did not change significantly, while γH2AX expression was increased. In contrast, after both sh-SNHG15 and DDP treatments for A549/CR cells, the expression of ECE2 was lower than that of DDP group, while the expression of γH2AX was significantly higher than that of DDP group, suggesting that sh-SNHG15 combined with DDP accelerated DNA damage in A549/CR cells (Fig. 5E, Supplementary Fig. 3). Afterwards, we examined the cell apoptosis in tumor sections by TUNEL assay and found that sh-SNHG15 was able to stimulate apoptosis in groups without or with DDP treatment (Fig. 5F). To sum up, SNHG15 could enhance DDP resistance in LUAD tissue in vivo.

## Discussion

Treatment outcomes for LUAD patients are severely restricted by chemoresistance, therefor it is vital to reveal the mechanism of LUAD resistance and explore new treatment strategies. SNHG15 can act on the biological functions of lung cancer and LUAD through different molecular mechanisms. Huang et al. [10] found that SNHG15 was highly expressed in LUAD, targeted miR-451 to regulate MDR-1, and promoted the resistance of LUAD cells to gefitinib. Cui et al. [33] found that SNHG15 was highly expressed in lung cancer tissues and affected viability, migration and other biological functions of lung cancer cells via miR-211-3p. We demonstrated that SNHG15 was markedly highly expressed in LUAD cells and tissues, as well as the LUAD-resistant cells. Knockdown of SNHG15 could attenuate DDP resistance in LUAD cells. The suppressive effect of silencing SNHG15 on DDP resistance could be reversed by ECE2 overexpression.

The investigation on the molecular mechanism of DDP resistance will accelerate the development of more effective targeted therapeutic strategies, and to tackle the problem of tumor drug resistance. We demonstrated that SNHG15 was highly-expressed in LUAD-resistant cells and knockdown of SNHG15 enhanced the DDP sensitivity of LUAD-resistant cells. Actually, previous studies reported that altered SNHG15 was correlated with chemoresistance. For instance, Zhang et al. [34] revealed that down-regulating SNHG15 attenuated the proliferation of osteosarcoma cells and enhanced their sensitivity to adriamycin. Qu et al. [12] reported that overexpressed SNHG15 accelerated epithelial ovarian cancer cell invasion, migration, and proliferation and could enhance the DDP resistance of tumor cells. Mi et al. [8] reported that SNHG15 strengthened DDP resistance in breast cancer by targeting miR-381. The above results suggested that SNHG15 might be an effective target for the adjuvant therapy for DDP-resistant patients.

Although we have clarified the effect of SNHG15 on DDP resistance, the molecular mechanism by which SNHG15 affects DDP resistance remains unclear. Thus, we further explored its downstream molecular mechanism. After the assays and bioinformatics analysis, we demonstrated that through recruiting E2F1, SNHG15 could affect the expression of downstream gene ECE2 to promote LUAD resistance. E2F family is formed by TFs related to cell cycle, in which E2F1 is included [35]. E2F1 was identified to be a key factor influencing cellular drug resistance. For instance, Xu et al. [36] claimed that up-regulated E2F1 suppressed ENZ and DTX resistance in prostate cancer cells. Yan et al. [37] demonstrated that overexpressed E2F1 stimulated multidrug resistance in gastric cancer, including DDP, adriamycin, and 5-FU. This study confirmed ECE2 as a downstream target gene of E2F1, while the effect of ECE2 on tumor progression was rarely illustrated. We found that ECE2 expression was notably high in LUAD-resistant cells, which was markedly suppressed through silencing SNHG15. Although we demonstrated that ECE2 could modulate DDP drug resistance, there is still no target drug for this gene. Therefore, this study can theoretically support the development of DDP therapeutic sensitization drugs.

The molecular mechanism of platinum-based chemotherapeutic agents is the formation of various cross-linked and mono-addition products through binding to DNA and non-DNA. Then they will stimulate the cytotoxicity through inducing apoptosis and inhibiting DNA replication [38]. It will be confirmed that they may develop resistance to DDP and survive in chemotherapy, if damage repair occurs in tumor cells [39]. Previous study suggested that lncRNA UCA1 accelerated proliferation of oral squamous cell carcinoma and enhanced drug resistance to DDP through stimulating DNA damage repair [40]. In breast cancer cells, lncMat2B induced DDP resistance through increasing DNA damage repair [41]. The above results presented that it was common that tumor cells induced DDP resistance by modulating DNA damage repair. In response to the above phenomenon, DDP combination regimens targeting tumor DNA damage repair are available. For example, inhibition of ATR with AZD6738 enhanced DDP-induced DNA damage in HNSCC cells, thereby improving the sensitivity of tumor cells to DDP [42]. Clinical studies reported that compared to chemotherapy regimens alone, combination regimens of PARP-targeted agents targeting DNA damage repair and DDP dramatically improved progression-free survival for breast and ovarian cancer patients [43]. In summary, targeting DNA damage to improve DDP sensitivity is a viable option for improving patient outcomes. In this study, SNHG15 was found to enhance DDP resistance by attenuating DNA damage, and combination therapies targeting SNHG15 may be a feasible option to improve DDP treatment outcomes.

In summary, we initially demonstrated the involvement of SNHG15 in the DDP resistance in LUAD both in vivo and in vitro via the E2F1/ECE2 axis. The SNHG15/E2F1/ECE2 axis may provide new perspectives for developing effective strategies to cope with DDP resistance in LUAD cells. However, this study still has some deficiencies, in terms of the lack of clinical study on SNHG15/E2F1/ECE2 axis and the verification of its capability for clinical application. This will be an important direction for our future research, and we will collect clinical samples for systematic analysis and investigation of this topic.

## Supplementary Information

**Additional file 1: Supplementary Fig. 1.** SNHG15/E2F1/ECE2 axis enhances DDP resistance in H1975/CR cells. (A) SNHG15 expression in H1975/CS and H1975/CR cells assessed by qRT-PCR. (B) IC50 values of DDP in H1975/CS and H1975/CR cells evaluated through CCK-8 assay. (C-D) The SNHG15 expression levels of H1975/CR cells transfected with sh-NC+oe-NC, sh-SNHG15+oe-NC, sh-NC+oe-ECE2 and sh-SNHG15+oe-ECE2 were detected by qRT-PCR and western blotWestern blot. (E) IC50 values of DDP transfected with sh-NC+oe-NC, sh-SNHG15+oe-NC, sh-NC+oe-ECE2 and sh-SNHG15+oe-ECE2 in H1975/CR cells were tested through CCK-8 assay. (F) DNA damage transfected with sh-NC+oe-NC, sh-SNHG15+oe-NC, sh-NC+oe-ECE2 and sh-SNHG15+oe-ECE2 in DDP-treated (3 µg/mL) H1975/CR cells were observed by comet assay. (Scale bar: 20 µm). (G) Immunofluorescence assay was performed to assess γH2AX production in DDP-treated (3 µg/mL) H1975/CR cells transfected with sh-NC+oe-NC, sh-SNHG15+oe-NC, sh-NC+oe-ECE2 and sh-SNHG15+oe-ECE2, and photographs were taken by using confocal microscopy. (Scale bar: 20 µm) (*/# indicates *P*<0.05)

**Additional file 2: Supplementary Fig. 2.** SNHG15/E2F1/ECE2 axis enhances DDP resistance in H1299/CR cells. (A) SNHG15 expression in H1299/CS and H1299/CR cells assessed by qRT-PCR. (B) IC50 values of DDP in H1299/CS and H1299/CR cells evaluated through CCK-8 assay. (C-D) The SNHG15 expression in H1299/CR cells transfected with sh-NC+oe-NC, sh-SNHG15+oe-NC, sh-NC+oe-ECE2 and sh-SNHG15+oe-ECE2 were determined by qRT-PCR and western blotWestern blot. (E) IC50 values of DDP in H1299/CR cells transfected with sh-NC+oe-NC, sh-SNHG15+oe-NC, sh-NC+oe-ECE2 and sh-SNHG15+oe-ECE2 were evaluated through CCK-8 assay. (F) DNA damage in DDP-treated (3 µg/mL) H1299/CR cells transfected with sh-NC+oe-NC, sh-SNHG15+oe-NC, sh-NC+oe-ECE2 and sh-SNHG15+oe-ECE2 were observed by comet assay. (Scale bar: 20 µm). (G) Immunofluorescence assay was performed to assess γH2AX production in DDP-treated (3 µg/mL) H1299/CR cells transfected with sh-NC+oe-NC, sh-SNHG15+oe-NC, sh-NC+oe-ECE2 and sh-SNHG15+oe-ECE2, and photographs were taken by using confocal microscopy. (Scale bar: 20 µm) (*/# indicates *P*<0.05).

**Additional file 3: Supplementary Fig. 3.** Determination of ECE2 and γH2AX in different transfection groups by IHC.

## Data Availability

The data used to support the findings of this study are included within the article.
