# Supplementary figures and images for: SNHG15 enhances cisplatin resistance in lung adenocarcinoma by affecting the DNA repair capacity of cancer cells
Source: Diagn Pathol. 2023 Mar 2;18:33. doi: 10.1186/s13000-023-01291-2 (PMC9979449; doi:10.1186/s13000-023-01291-2)

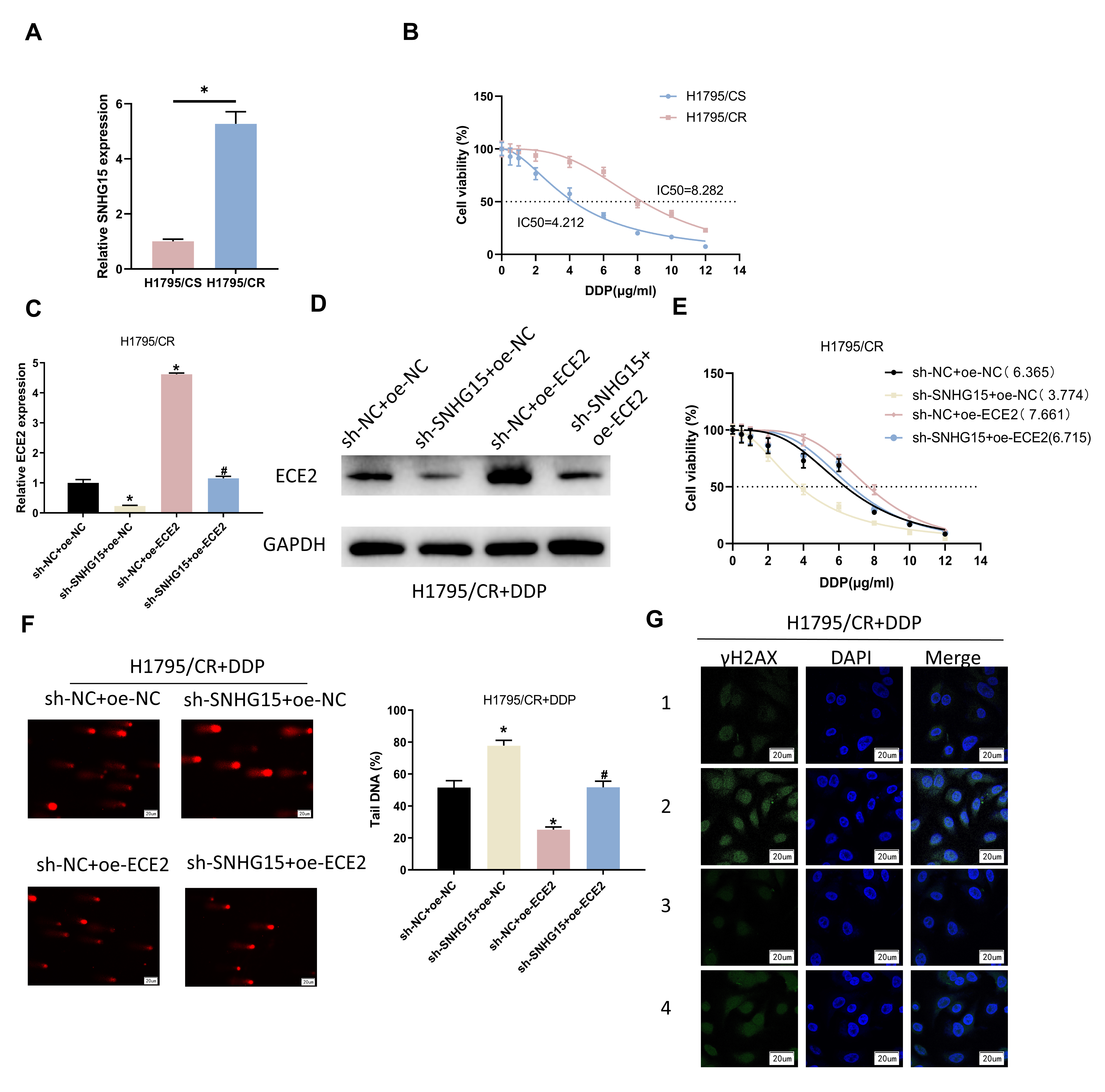

Supplement: Supplementary file 1 — Additional file 1: Supplementary Fig. 1. SNHG15/E2F1/ECE2 axis enhances DDP resistance in H1975/CR cells. (A) SNHG15 expression in H1975/CS and H1975/CR cells assessed by qRT-PCR. (B) IC50 values of DDP in H1975/CS and H1975/CR cells evaluated through CCK-8 assay. (C-D) The SNHG15 expression levels of H1975/CR cells transfected with sh-NC+oe-NC, sh-SNHG15+oe-NC, sh-NC+oe-ECE2 and sh-SNHG15+oe-ECE2 were detected by qRT-PCR and western blotWestern blot. (E) IC50 values of DDP transfected with sh-NC+oe-NC, sh-SNHG15+oe-NC, sh-NC+oe-ECE2 and sh-SNHG15+oe-ECE2 in H1975/CR cells were tested through CCK-8 assay. (F) DNA damage transfected with sh-NC+oe-NC, sh-SNHG15+oe-NC, sh-NC+oe-ECE2 and sh-SNHG15+oe-ECE2 in DDP-treated (3 µg/mL) H1975/CR cells were observed by comet assay. (Scale bar: 20 µm). (G) Immunofluorescence assay was performed to assess γH2AX production in DDP-treated (3 µg/mL) H1975/CR cells transfected with sh-NC+oe-NC, sh-SNHG15+oe-NC, sh-NC+oe-ECE2 and sh-SNHG15+oe-ECE2, and photographs were taken by using confocal microscopy. (Scale bar: 20 µm) (*/# indicates P<0.05) [file 13000_2023_1291_MOESM1_ESM.tif]

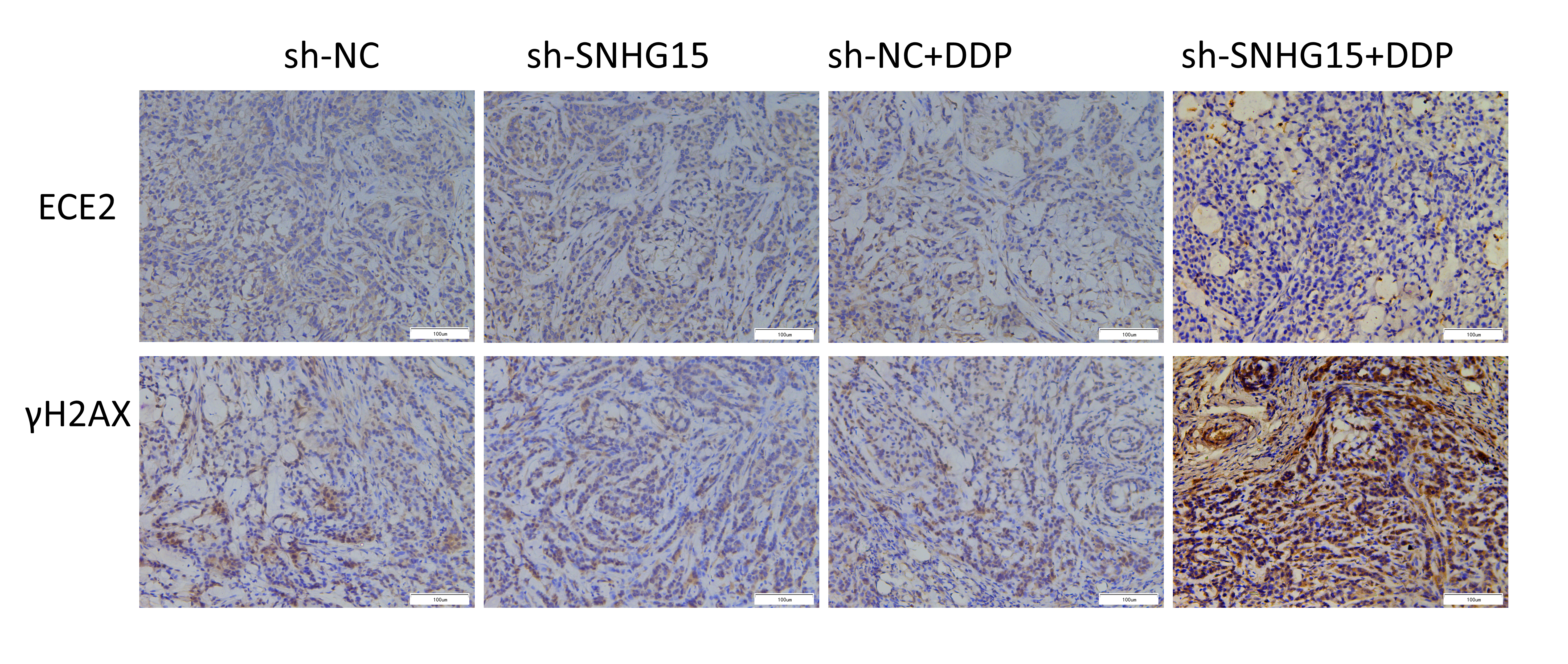

Supplement: Supplementary file 3 — Additional file 3: Supplementary Fig. 3. Determination of ECE2 and γH2AX in different transfection groups by IHC. [file 13000_2023_1291_MOESM3_ESM.tif]
